# Ultrasound Imaging in Predicting the Autograft Size in Anterior Cruciate Ligament Reconstruction: A Systematic Review and Meta-Analysis

**DOI:** 10.3390/jcm11133876

**Published:** 2022-07-04

**Authors:** Tsung-Min Lee, Wei-Ting Wu, Yi-Hsiang Chiu, Ke-Vin Chang, Levent Özçakar

**Affiliations:** 1 Department of Physical Medicine and Rehabilitation, National Taiwan University College of Medicine, Taipei 10051, Taiwan; braden2011@hotmail.com.tw (T.-M.L.); wwtaustin@yahoo.com.tw (W.-T.W.); chiu19910703@gmail.com (Y.-H.C.); 2 Department of Physical Medicine and Rehabilitation, National Taiwan University Hospital, Bei-Hu Branch, Taipei 10845, Taiwan; 3 Center for Regional Anesthesia and Pain Medicine, Wang-Fang Hospital, Taipei Medical University, Taipei 11031, Taiwan; 4 Department of Physical and Rehabilitation Medicine, Hacettepe University Medical School, Ankara 06230, Turkey; lozcakar@yahoo.com

**Keywords:** knee, sports injury, anterior cruciate ligament, revision surgery, ultrasonography

## Abstract

Anterior cruciate ligament (ACL) reconstruction is widely used to restore knee stability after injury, but the risk of revision surgery increases when the autograft size is inadequate. Ultrasound (US) measurements of preoperative target tendons have been applied to predict the intraoperative autograft size, with various outcomes across different studies. This systematic review and meta-analysis aimed to summarize the evidence and investigate the usefulness of US in predicting autograft size. Electronic databases were searched for relevant studies from inception to 19 January 2022. The primary outcome was the correlation between the preoperative US measurements of donor tendons and intraoperative autograft size. The secondary outcomes encompassed the predictive performance of US for autograft size and the comparison between US and magnetic resonance imaging (MRI) for preoperative tendon measurements. Nine studies, comprising 249 patients, were enrolled. The preoperative US measurements of the donor tendons demonstrated a significant positive correlation with their intraoperative autograft diameter, with a pooled correlation coefficient of 0.443 (95% confidence interval [CI], 0.266–0.591, *p* < 0.001) for the gracilis and semitendinosus autograft, 0.525 (95% CI, 0.114–0.783, *p* = 0.015) for the semitendinosus autograft, and 0.475 (95% CI, 0.187–0.687, *p* = 0.002) for the gracilis autograft. The pooled sensitivity and specificity of US imaging in predicting the autograft diameter were 0.83 (95% CI 0.57–0.95) and 0.70 (95% CI, 0.36–0.91), respectively. Moreover, no significant differences were observed between US and MRI measurements in predicting the sizes of the gracilis and semitendinosus autografts. Preoperative US measurements of the target tendons were moderately correlated with the intraoperative autograft size. US imaging has a discriminative performance similar to that of MRI in predicting the autograft size. A standardized US scanning protocol is needed for future studies to minimize the variations in tendon measurements across different investigators and increase the comparability of US imaging with intraoperative findings.

## 1. Introduction

Anterior cruciate ligament (ACL) injury is one of the most prevalent sports injuries, with an incidence ranging from 36.9 to 60.9 per 100,000 person–years [1,2]. Lateral pivoting, landing, and deceleration are maneuvers that are highly associated with ACL injuries during sports play [3]. Conservative management for ACL ruptures includes physiotherapy, supportive bracing, and physical activity modification [4]. Nonetheless, when patients are athletes with persistent disability after nonoperative treatments, surgical management is needed to restore knee joint stability [4].

ACL reconstruction has been widely used, with a satisfactory outcome rate between 75% and 97% [5]. Its benefits include reducing the risk of subluxation and decreasing the incidence of early posttraumatic osteoarthritis [6]. Herewith, up to 8% of patients have been reported to undergo revision surgery following ACL reconstruction [7], whereby the risk factors include low patient-reported functional outcomes and radiographic signs of tibiofemoral osteoarthritis [8]. The increased rate of revision surgery following ACL reconstruction has been associated with a hamstring autograft diameter of <8 mm [9,10]. In this regard, predicting the autograft size before ACL reconstruction appears to be critical for the surgeon [11].

Various imaging techniques, i.e., three-dimensional computed tomography (3DCT), magnetic resonance imaging (MRI), and ultrasound (US), have been proposed for predicting the autograft size in different studies. For instance, the 3DCT-measured semitendinosus tendon length showed a high positive correlation with graft length [12]. The MRI-measured tendon cross-sectional area (CSA) yielded a better predictive value than that of the MRI-measured tendon diameter in estimating the hamstring graft size [13]. US has advantages over the aforementioned methods such as real-time image acquisition, low cost, zero ionizing radiation, and high resolution for superficial structures [14]. However, some studies demonstrated moderate correlations between the US-measured CSA of the donor tendon and its autograft diameter [14,15,16,17,18,19,20], whereas others did not [21,22].

To date, reviews quantifying the effectiveness of the use of preoperative US imaging in predicting intraoperative autograft size are nonexistent [23]. Therefore, this meta-analysis aimed to investigate the effectiveness of US imaging in predicting the autograft size in patients receiving ACL reconstruction.

## 2. Methods

### 2.1. Protocol Registration

This systematic review and meta-analysis was based on a preplanned protocol constructed in accordance with the standard Preferred Reporting Items for Systematic Reviews and Meta-Analysis (PRISMA) guidelines [24]. The details are provided in the PRISMA checklist (supplemental material). The protocol was prospectively registered on inplasy.com on 23 January 2022 (INPLASY202210114).

### 2.2. Data Sources and Search Strategy

Five electronic databases, including PubMed, Cochrane CENTRAL, Embase, Clincial.gov, and Web of Science, were searched for relevant studies from inception to 19 January 2022 without language restrictions. The manual retrieval of additional studies was performed using relevant narratives and systemic reviews. The PICO question was constructed as follows: P, patients undergoing ACL reconstruction using the autograft; I, preoperative graft size assessment on US imaging; C, intraoperative graft size; O, predictive performance of the graft size. The following strategies were used for the literature search: ((ultrasound) OR (sonography) OR (echography) OR (ultrasonography)) AND ((graft size) OR (graft assessment)) AND ((anterior cruciate ligament surgery) OR (anterior cruciate ligament reconstruction)). The complete search strategy is presented in the Supplementary Information.

### 2.3. Inclusion and Exclusion Criteria

The studies were included if they (1) were an original research work investigating ACL reconstruction using an autograft, (2) were using US imaging for the preoperative assessment of the donor tendon, (3) had documentation of the intraoperative autograft size, and (4) were human studies.

The exclusion criteria were as follows: (1) case reports/series, reviews, study protocols, editorials, or commentaries; (2) preoperative autograft assessment using CT or MRI only; (3) lack of information regarding the intraoperative graft size; and (4) studies without available data either for the correlations between US measurements and intraoperative autograft size or for the accuracy of US imaging in predicting the adequacy of the autograft size.

### 2.4. Data Extraction

Following the literature search of the electronic databases, two authors scrutinized the abstracts from the retrieved articles independently. If there was disagreement between the two reviewers regarding the selected articles, a decision was made through discussion or the corresponding author decided. Full texts of the eligible articles were subsequently downloaded, and data were extracted using a standardized form in Microsoft Excel 2016 (Microsoft Corporation 2016). The excerpted information consisted of the name of the first author, year of publication, study design, autograft choice, age and sex of participants, US settings, surgical procedures for ACL reconstruction, interval between US measurement and ACL reconstruction, outcome of interest, and reference standard. 

### 2.5. Outcomes

The primary outcome of the study was the correlation between the preoperative US measurements of the donor tendon and intraoperative autograft diameter. The secondary outcomes included the prediction of the size adequacy of the autograft using US imaging and the comparison of US and MRI measurements concerning the preoperative size.

### 2.6. Study Quality Assessment

The Quality Assessment of Diagnostic Accuracy Studies (QUADAS)-2 was used to assess the quality of studies included in the meta-analysis [25]. Accordingly, each article was evaluated for risks of bias in four domains. A low risk of bias in each domain was defined as follows: (1) patient selection—the study excluded patients who could introduce spectrum bias (the performance of a diagnostic test varied according to differences in disease severity); (2) index test—the preoperative US measurements were interpreted without knowing the results of the intraoperative autograft size; (3) reference standard—the intraoperative autograft diameter was measured using calibrated holes; and (4) flow and timing—all patients received preoperative US and intraoperative measurements of the autograft size.

Each article was evaluated for its applicability to the research question. Based on the domains of patient selection, index test, and reference standard, we defined low concern of applicability as follows: (1) patient selection—patients presented to the health care setting with ACL injuries who were scheduled for ACL reconstruction; (2) index test—preoperative tendon measurement was performed using US imaging; and (3) reference standard—the intraoperative autograft size was measured.

### 2.7. Statistical Analysis

Correlations between the size of the donor tendon measured by US/MRI and its autograft diameter were summarized using the Hedges–Olkin method based on the Fisher Z transformation of the variables [26]. The weighted mean difference was used to investigate the discrepancy between the US and MRI measurements of the donor tendon CSA [27]. The performance of predicting the size adequacy of the autograft was evaluated by the average sensitivity, specificity, positive/negative likelihood ratios, and diagnostic odds ratio using a bivariate random-effects model [28,29]. The summary receiver operating characteristic (SROC) curve was applied to pool and inspect the predictive performance of each enrolled study as well as to obtain the area under the curve [30]. The size/extent of variability of the target parameters across the included studies was determined using *I*^2^, which denotes the proportion of variation across studies that is caused by heterogeneity rather than chance. An *I*^2^ > 50% was considered significant [31]. Funnel plots were built to examine the publication bias, which was also determined by Egger’s test for continuous variables and Deeks’ funnel plot asymmetry test for diagnostic accuracy [32,33]. All statistical analyses were conducted using Stata (StataCorp 2015, Stata Statistical Software: Release 14, StataCorp LP, College Station, TX, USA) and Comprehensive Meta-analysis Software, version 3 (Biostat, Englewood, NJ, USA); *p* < 0.05 was considered statistically significant. Meta-DiSc (version 1.4, Hospital Ramon y Cajal and Universidad Complutense de, Madrid, Spain) was specifically used to analyze the data for the predictive performance when the number of available studies was fewer than four.

## 3. Results

### 3.1. Literature Search

**Figure 1:**
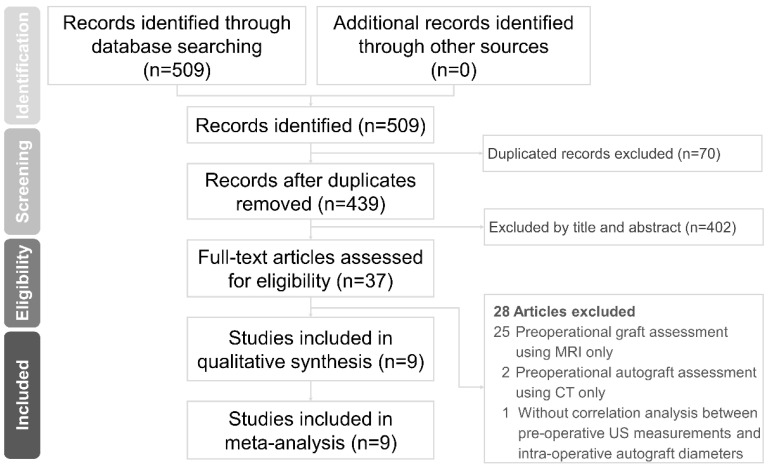
Flow diagram of the literature search based on the Preferred Reporting Items for Systematic Reviews and Meta Analyses (PRISMA) guidelines. MRI, magnetic resonance imaging; CT, computed tomography; US, ultrasound.

A total of 509 articles were initially accessed from the electronic databases. After eliminating duplicates, 439 articles were left, and 37 were related to our topic (based on their titles/abstracts). After reading their full texts, nine articles met the inclusion criteria and were enrolled in the meta-analysis [14,15,16,17,18,19,20,21,22]. The reason for article exclusion is summarized in Supplementary Table S1. A flow diagram of the literature search is shown in Figure 1.

### 3.2. Study Characteristics

A total of 1 cross-sectional [21] and 8 cohort studies [14,15,16,17,18,19,20,22] comprising 249 participants undergoing ACL reconstruction were included. The mean age of the patients ranged from 19.9 to 32 years. The study characteristics are summarized in Table 1. Regarding the selection of autografts, seven studies used the four-strand semitendinosus and gracilis tendons [14,15,16,17,19,21,22], one study used the four-strand semitendinosus tendon [18], and one study used the quadriceps tendon [20]. Regarding the preoperative US assessment of the donor tendons, eight studies [14,15,17,18,19,20,21,22] provided the CSA, and one study [16] measured the diameter. Other than US, three studies [14,15,20] used additional MRI to evaluate the preoperative autograft size. Regarding the size adequacy of the intraoperative autograft diameter, the cutoff values were 8 mm in seven studies [15,16,18,19,20,21,22] and 7.5 mm [14] and 7 mm [17] in the other two studies.

### 3.3. Quality Assessment

Table 2 illustrates the methodological analysis of the included studies based on QUADAS-2. All studies showed a low risk of bias regarding the domains of patient selection, index test, and reference standard. Three studies [14,16,18] showed a high risk of bias in the domain of flow and timing due to an unclear interval between the index test and the reference standard. All studies showed low concern regarding applicability.

### 3.4. Outcome

#### 3.4.1. Correlations between Preoperative US and Intraoperative Autograft Measurements

Preoperative US measurements (mainly CSA) of the gracilis and semitendinosus tendons demonstrated a significant positive correlation with the intraoperative autograft diameter based on eight enrolled studies [14,15,16,17,18,19,21,22], with a pooled correlation coefficient of 0.443 (95% CI, 0.266–0.591, *p* < 0.001; *I*^2^ = 50.88%). No significant publication bias was detected when examining the symmetry of the effect sizes on the funnel plot and hypothesis testing using Egger’s test (*p* = 0.709) (Supplementary Figure S1).

**Figure 2:**
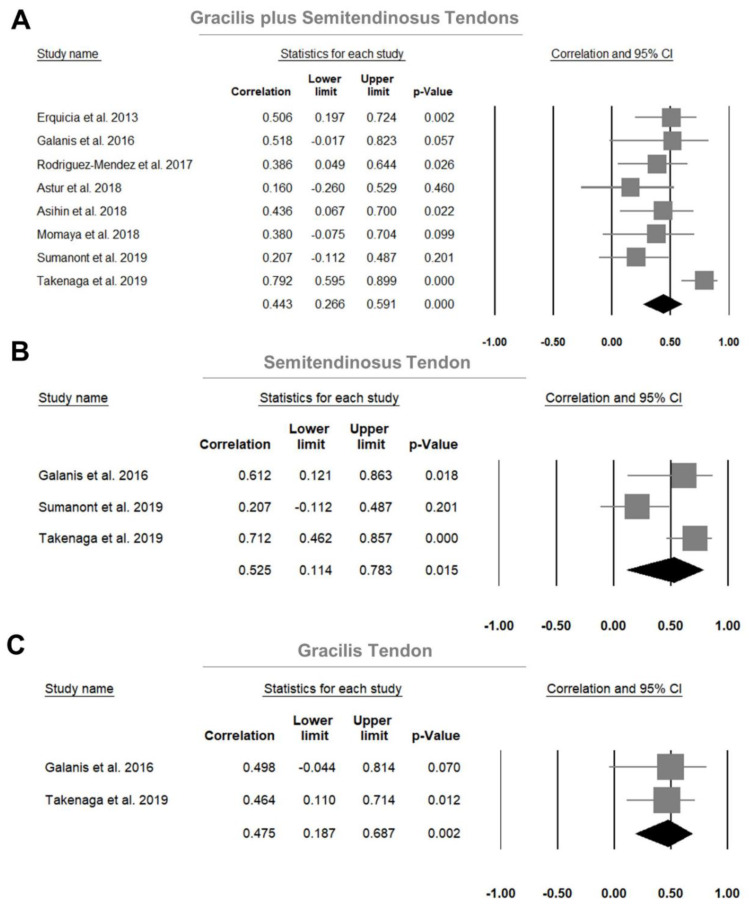
Forest plots of the summarized correlations between the ultrasound measurements and the intra-operative autograft diameter for (**A**) the gracilis plus semitendinosus tendons, (**B**) the semitendinosus tendon and (**C**) the gracilis tendon. CI, confidential interval.

A significant positive correlation (pooled correlation coefficient, 0.525; 95% CI, 0.114–0.783, *p* = 0.015; *I*^2^ = 72.99%) was also identified between the preoperative US measurements (CSA) of the semitendinosus tendons and the intraoperative autograft diameter from the three included studies [14,18,19]. Similarly, a significant positive correlation existed between the preoperative US measurements (CSA) of the gracilis tendon and the autograft diameter based on two enrolled studies [14,19], with a pooled correlation coefficient of 0.475 (95% CI, 0.187–0.687, *p* = 0.002; *I*^2^ < 0.001). Forest plots of the aforementioned correlations are shown in Figure 2.

#### 3.4.2. US Imaging in Predicting the Size Adequacy of the Autograft

**Figure 3:**
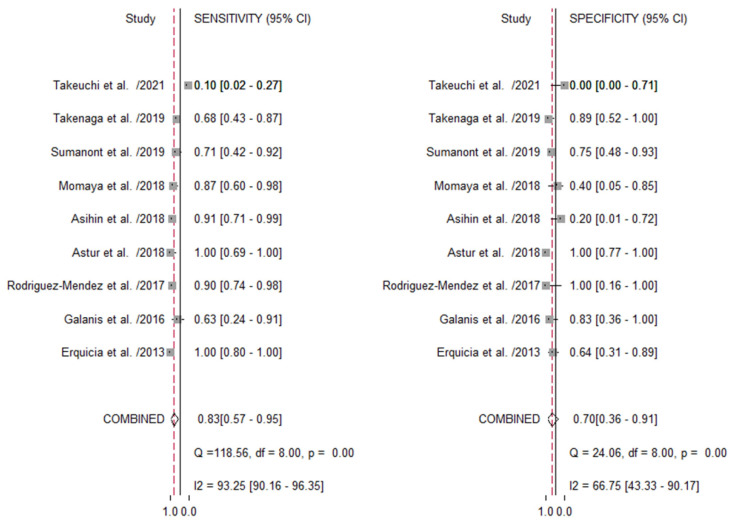
Forest plot of the summarized sensitivity and specificity of ultrasound imagining for predicting the autograft size inadequacy. CI, confidential interval.

The average sensitivity and specificity in predicting the size adequacy of the autograft using US imaging was 0.83 (95% CI, 0.57–0.95, *p* < 0.001; *I*^2^ = 93.25%) and 0.70 (95% CI, 0.36–0.91, *p* < 0.001; *I*^2^= 66.75), respectively (Figure 3).

The pooled positive likelihood, negative likelihood, and diagnostic odds ratios were 2.80 (95% CI, 0.90–8.4), 0.24 (95% CI, 0.06–0.91), and 12 (95% CI, 1–118), respectively. Based on the SROC curve (Supplementary Figure S2), the area under the curve was 0.84 (95% CI, 0.81–0.87). The Deeks’ funnel plot asymmetry test revealed no significant evidence of publication bias (*p* = 0.21) (Supplementary Figure S3).

#### 3.4.3. Comparison between US and MRI Measurements in Predicting the Autograft Size

**Figure 4:**
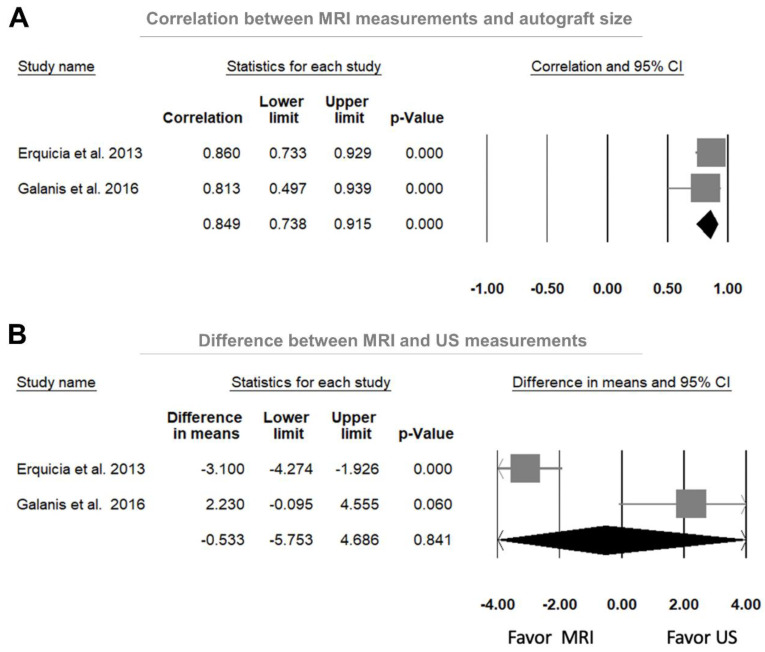
Forest plots of (**A**) the correlations between the magnetic resonance imaging (MRI)-measured cross-sectional area (CSA) and the intra-operative graft diameter and (**B**) the weighted mean difference between the MRI-measured and the ultrasound (US)-measured CSA. CI, confidential interval.

A significant positive correlation was observed between the MRI-measured CSA and the autograft diameter of the gracilis and semitendinosus tendons based on the two included studies [14,15], with a pooled correlation coefficient of 0.849 (95% CI, 0.738–0.915, *p* < 0.001; *I*^2^ = 94.21%; Figure 4A). In addition, no significant difference was found between the MRI- and US-measured CSA of the gracilis and semitendinosus tendons based on the two included studies [14,15], with a weighted median difference of −0.533 mm^2^ (95% CI, −5.753–4.686, *p* = 0.841; *I*^2^ = 94.21%; Figure 4B).

Further, we additionally examined the predictive performance of MRI for autograft diameter adequacy from three included studies [14,15,20]. The average sensitivity and specificity were 0.97 (95% CI, 0.87–0.99; *I*^2^ < 0.01%) and 0.53 (95% CI, 0.34–0.70; *I*^2^ ≤ 0.01%), respectively. The pooled positive likelihood, negative likelihood and diagnostic odds ratios were 1.50 (95% CI, 0.55–4.05), 0.23 (95% CI, 0.03–1.48), and 10.45 (95% CI, 0.28–389.71), respectively. The corresponding area under the curve was not computed because of the inadequacy of the number of studies with available data.

## 4. Discussion

This meta-analysis unmasked several important findings. First, there was a moderate correlation between the preoperative US measurements of the donor tendons and the intraoperative autograft size. Second, the average sensitivity and specificity in predicting the adequacy of autograft size reached 0.83 and 0.70, respectively. Third, no significant difference was found between the US and MRI measurements of the donor tendon size.

Some factors need to be considered before interpreting the correlation coefficients between the US measurements of the donor tendon and the autograft diameter. The size of the autograft could only be represented by its diameter because tendon integrity is needed for ACL reconstruction. The transection of the target tendon to obtain the CSA is not practical. However, as the target tendon may not be in a circular or symmetrically oval shape, it is challenging to define the diameter on US images. Unlike the diameter, the CSA can be measured by tracking the border of the tendon. It better represents the tendon size and can serve as an optimal surrogate for predicting the autograft diameter. Therefore, the majority of the included studies employed the CSA to estimate the autograft size. In 2012, Mukaka et al. [34] defined a correlation coefficient between 0.5 and 0.7 to indicate a moderate degree of correlation. In our meta-analysis, the point estimate of the pooled correlation coefficients ranged between 0.443 and 0.525, indicating a low to moderate correlation between the US-measured tendon size and autograft diameter. Since the measurement is two-dimensional for the CSA of the donor tendons and one-dimensional for the intraoperative autograft diameter, the data discrepancy may cause lower correlations than anticipated.

Most previous studies have suggested that the diameter of donor tendons should be >7 mm to avoid graft failure [35]. Similarly, recent large-scale studies have reported an increased revision rate if the hamstring autograft size was <8 mm. In 2013, Mariscalco et al. [36] reported that among 320 participants, 15.3% with autografts <8 mm needed revision surgery. In 2021, Alkhalaf et al. [37] enrolled 782 cases and found that patients with an autograft size <8 mm were 7.2 times more likely to experience ACL reconstruction failure. In most of our included studies, 8 mm was treated as the threshold of autograft size inadequacy, although the cutoff points of US-measured CSA varied significantly. Our meta-analysis revealed that the pooled sensitivity and specificity in predicting autograft size inadequacy were 0.83 and 0.70, respectively. The point estimate of the diagnostic odds ratio for US imaging could reach 12, indicating its ability to discriminate participants with and without an inadequate autograft size.

Our study revealed that the pooled diagnostic odds ratio for MRI was 10.45, indicating the usefulness of MRI in predicting the autograft size. In 2016, Grawe et al. [38] reported that a CSA of the donor tendon >22 mm^2^ could reliably predict a graft diameter >8 mm. In 2017, Leiter et al. [39] found that the CSA of the semitendinosus and gracilis tendons measured on MRI was a good surrogate for predicting the autograft diameter. Our meta-analysis also revealed no significant differences between the US- and MRI-measured CSA values. In other words, the predictive performance appears to be similar between the two imaging modalities.

According to our results, the pooled specificity of US imaging is lower than the pooled sensitivity in discriminating the size inadequacy of the autograft (0.70 vs. 0.83). This finding suggests that the ability of US imaging to detect donor tendon sizes lower than the threshold (specificity) was not as good as its ability to identify a tendon size higher than the cutoff point (sensitivity). As size inadequacy is associated with an increased risk of autograft failure, specificity would be more important than sensitivity in clinical practice.

The lower specificity of US imaging may be attributed to several factors. First, the US scanning protocols varied across different studies. There was a noticeable difference in donor tendon size between the myotendinous junction and distal attachment levels. As the sizes of the hamstring tendons are not the same at different levels [40], accurate comparison is not possible on this basis. Second, it could be challenging to differentiate the paratenon from the tendon tissues using US imaging. This may lead to variations in the estimated autograft size as the surrounding connective tissues need to be excised during ACL reconstruction.

A recent ultrasound study [41] showed that the US-measured CSA was highly correlated with that calculated under MRI, with intra-class correlation coefficients ranging from 0.882 to 0.996. A standardized level of measurements is prerequisite for reaching such satisfactory reliability, which seems to be lacking in our included studies. Furthermore, whether the transducer was perpendicular to the tendon or the examined knees were extended or flexed at a certain angle significantly affects the comparability. Among our enrolled articles, we also identified no details regarding the transducer used for image acquisition by Momaya et al. [22] and Sumanont et al. [18], which also made their work not as reliable as others.

However, although US imaging might be limited by its ability to detect the size inadequacy of the autograft, it is still beneficial to perform US scanning of the target tendon before surgery. Because most patients traumatized their ACL due to sport injury, their hamstring or patellar tendons might be collaterally damaged. US imaging would be helpful to check whether the donor tendons have scars or tears, which might affect the durability of the autograft.

## 5. Limitations

This study has several limitations. First, the interval between the US examination and the operation was unclear in some of the included studies. Tendon size may vary at different time points. Second, the number of participants in each study was relatively small, which limits the power of the present meta-analysis. Third, none of the included studies stratified the patients’ ages into different groups for analysis. Furthermore, most of the recruited participants were relatively young, possibly due to injury during sporting. It may be difficult to generalize our study results to older populations receiving ACL reconstruction. Future studies should investigate the influence of age regarding the US measurements of target tendons.

## 6. Conclusions

This meta-analysis indicated that preoperative US measurements of donor tendons could be moderately correlated with the intraoperative autograft size. Moreover, US and MRI had similar discriminative performance with regard to the prediction of autograft size inadequacy. However, US measurements must be meticulous and comparative; otherwise, the benefits of US imaging would be lost. Standardized scanning protocols are needed for future studies to minimize the variations in tendon measurements across different investigators and increase the comparability of US imaging with intraoperative findings. As there was only a small number of included studies in this meta-analysis whose statistics were relatively descriptive, the application of US imaging for the prediction of the autograft size should be exercised with caution in clinical practice.

## Figures and Tables

**Table 1:** Summary of the retrieved studies investigating the predictive values of ultrasound imaging for autograft size in anterior cruciate ligament reconstruction.

Study, Year	Study Design	Autograft	Age	M/F	Ultrasound Setting		Surgical Procedure	Interval US—OP	Outcome	Reference Standard
					Manufacturer, Transducer Frequency, CSA Measurement	Probe Position, Examinee Posture, Site of US Measurements				
Erquicia, 2013 [15]	Prospective cohort	4S-GST	32 (16–59) ^†^	25/8	LOGIQe, GE Healthcare Linear array probe, 7–12 MHz, ellipse tool	NA, prone, knee flexion 90°, proximal to the medial joint line	GT, ST harvested GT, ST paired, closed-hole sizing block	15 days	CSA: GT (US, MRI), ST (US, MRI), GT + ST (US, MRI) Diameter: 4S-GST (OP) No inter-rater, intra-rater reliability	Autograft diameter
Galanis, 2016 [14]	Prospective cohort	4S-GST	31.14 ± 3.11 *	14/0	Siemens Acuson S2000 Linear array probe, 10 MHz, ellipse or dotted line tool	Perpendicular to the tendon, prone, knee flexion 30°, near the widest point of the medial femoral epicondyle	GT, ST tendons harvested GT, ST paired, closed-hole sizing block	NA	CSA: GT + ST (US, MRI), ST (US, MRI), GT (US, MRI) Diameter: 4S-GST (OP), ST (US, MRI), GT (US, MRI) Inter-rater and intra-rater reliability	Autograft diameter
Rodriguez-Mendez, 2017 [16]	Prospective cohort	4S-GST	(16–43) ^†^	33/0	Siemens Acuson S2000 Linear array probe, 14 MHz, NA	Perpendicular to the tendon, prone, knee flexion 0°, posterior medial of proximal tibia with widest zone	GT, ST tendons harvested GT, ST folded a quadruple tendon	NA	Diameter: GT + ST (US), GT (US, OP), ST (US, OP), 4S-GST (OP) Length: 4S-GST (OP), ST (OP), GT (OP) No inter-rater, intra-rater reliability	Autograft diameter
Astur, 2018 [21]	Cross-sectional	4S-GST	24.8 ± 8.4 *	19/5	Logic P6 device, 7–11 MHz, NA	NA, ventral recumbent, the articular line	GT, ST tendons harvested ST, GT folded in half to form a quadruple graft	7 days	CSA: GT + ST (US) Diameter: GT (US), ST (US), 4S-GST (OP) No inter-rater, intra-rater reliability	Autograft diameter
Asihin, 2018 [17]	Prospective cohort	4S-GST	28.48 ± 6.0 *	23/4	Philips HD11 XE Linear array probe, 5–12 MHz, ellipse tool	NA, prone with knee flexion in 30°, the medial joint line	GT, ST harvested with a closed-end tendon harvester	1 day	CSA: ST + GT (US) Diameter: 4S-GST (OP) No inter-rater, intra-rater reliability	Autograft diameter
Momaya, 2018 [22]	Prospective cohort	4S-GST	22.8 ± 6.6 *	10/10	Fujifilm SonoSite, NA, NA	NA, prone with knee flexion in 30°	GT, ST harvested with a closed-loop tendon stripper	14 days	CSA: ST + GT (US) Diameter: 4S-GST (OP) Inter-rater, intra-rater reliability	Autograft diameter
Sumanont, 2019 [18]	Prospective cohort	4S-ST	29.3 ± 9.6 *	37/3	NA, NA, NA	NA, supine with knee flexion in 30°, the posterior medial aspect of the knee joint	ST harvested with a closed tendon stripper	NA	Diameter: ST (US, OP), 4S-ST (OP) Length: ST (US) CSA: ST (US) Inter-rater, intra-rater reliability	Autograft diameter
Takenaga, 2019 [19]	Prospective cohort	4S-GST	21.9 ± 8.6 *	11/17	Medicine RS80 Prestige linear-array probe, 4–18 MHz, freehand tracing	NA, supine with the hip in maximal ER and the knee in flexion 20°, the myotendinous junction of the sartorius muscle	GT, ST harvested with tendon stripper, suturing the distal end of tendon	11.3 ± 9.9 days *	CSA: GT + ST (US), ST (US), GT (US) Thickness: GT (US), ST (US) Width: GT (US), ST (US) Diameter: 4S-GST (OP), 2GT (OP), 2ST (OP) Inter-rater, intra-rater reliability	Autograft diameter
Takeuchi, 2021 [20]	Prospective cohort	QT	19.9 ± 5.0 *	18/12	Medicine RS80 Prestige linear-array probe, 4–18 MHz, NA	Perpendicular to the tendon, supine with the knee flexion in 20°, anterior knee proximal to the superior pole of the patella at a distance of 15 mm & 30 mm	QT harvested	17.9 ± 22.1 days *	CSA: QT (US, MRI) Diameter: QT (OP), QT (US, MRI) Inter-rater, intra-rater reliability	Autograft diameter

* mean ± standard deviation. ^†^ minimum to maximum. Abbreviations: CSA, cross-sectional area; ER, external rotation; GT, gracilis tendon; MHZ, megahertz; mm, millimeter; MRI, magnetic resonance imaging; NA, not applicable; OP, operation; QT, quadriceps tendon; ST, semitendinosus tendon; US, ultrasound; US-OP, interval between the ultrasound measurement and the anterior cruciate ligament reconstruction; 2GT, doubled gracilis tendon; 2ST, doubled semitendinosus tendon; 4S-GST, 4-strand gracilis plus semitendinosus tendon; 4S-ST, 4-strand semitendinosus tendon.

**Table 2:** The methodological quality of the included studies assessed by QUADAS-2.

	Risk of Bias				Applicability Concerns		
Study	Patient Selection	Index Test (US Measurement)	Reference Standard (Autograft Size)	Flow and Timing	Patient Selection	Index Test (US Measurement)	Reference Standard (Autograft Size)
Erquicia, 2013 [15]	Low	Low	Low	Low	Low	Low	Low
Galanis, 2016 [14]	Low	Low	Low	High	Low	Low	Low
Rodriguez-Mendez, 2017 [16]	Low	Low	Low	High	Low	Low	Low
Astur, 2018 [21]	Low	Low	Low	Low	Low	Low	Low
Asihin, 2018 [17]	Low	Low	Low	Low	Low	Low	Low
Momaya, 2018 [22]	Low	Low	Low	Low	Low	Low	Low
Sumanont, 2019 [18]	Low	Low	Low	High	Low	Low	Low
Takenaga, 2019 [19]	Low	Low	Low	Low	Low	Low	Low
Takeuchi, 2021 [20]	Low	Low	Low	Low	Low	Low	Low

Abbreviations: US, ultrasound; QUADAS, Quality Assessment of Diagnostic Accuracy Studies.

## Data Availability

The datasets used and/or analyzed during the current study are available from the corresponding author on reasonable request: contact Dr. Ke-Vin Chang (kvchang011@gmail.com).

## Supplementary Materials

The following supporting information can be downloaded at: https://www.mdpi.com/article/10.3390/jcm11133876/s1. Supplement Method. Table S1. Excluded studies and reasons [12,13,38,39,42,43,44,45,46,47,48,49,50,51,52,53,54,55,56,57,58,59,60,61,62,63,64,65]. Figure S1. Funnel plot for ultrasound measurements of the donor tendons in prediction of the autograft size among the included studies. Figure S2. The summary receiver operating curve (SROC) curve of ultrasound imaging for predicting size inadequacy of the autografts. Figure S3. Deek’s funnel plot for the assessment of potential publication bias of the included studies. Supplement PRISMA Checklist [66].
