# Supplementary material for: Ultrasound Imaging in Predicting the Autograft Size in Anterior Cruciate Ligament Reconstruction: A Systematic Review and Meta-Analysis
Source: J Clin Med. 2022 Jul 4;11(13):3876. doi: 10.3390/jcm11133876 (PMC9267791; doi:10.3390/jcm11133876)
Supplement: Supplementary file 1 [file jcm-11-03876-s001.zip › jcm-1765873 Supplemental-Material.pdf]

Supplement Materials:

Supplement Method

Supplement Table 1

Supplement Figures S1-S3

Supplement PRISMA Checklist

Supplement Method

Search strategy:

PubMed (Updated on January 19, 2022)

1. ultrasound OR sonography OR echography OR ultrasonography: 1,814,077
2. graft size OR graft assessment: 170,535
3. anterior cruciate ligament surgery OR anterior cruciate ligament reconstruction: 20,649
4. #1 AND #2 AND #3: 414

Filter: NA

History and Search Details — Download — Delete

Search	Actions	Details	Query	Results	Time
#4	•••	>	Search: **#1 AND #2 AND #3**	414	06:30:35
#3	•••	>	Search: **Anterior Cruciate ligament surgery OR Anterior Cruciate ligament reconstruction**	20,649	06:30:00
#2	•••	>	Search: **graft size OR graft assessment**	170,535	06:29:42
#1	•••	>	Search: **ultrasound OR sonography OR echography OR ultrasonography**	1,814,077	06:23:42

Showing 1 to 4 of 4 entries

EMBASE (Updated on January 19, 2022)

1. ultrasound OR sonography OR echography OR ultrasonography = #1 = 898,262
2. graft size OR graft assessment = #4 = 111,364
3. anterior cruciate ligament surgery OR anterior cruciate ligament reconstruction = #7 = 26,340
4. #1 AND #4 AND #7: 47

Filter: NA

#8	#1 AND #4 AND #7	47
#7	#5 OR #6	26,340
#6	'anterior cruciate ligament reconstruction'	15,418
#5	anterior AND cruciate AND ligament AND surgery	24,418
#4	#2 OR #3	111,364
#3	graft AND assessment	84,553
#2	graft AND size	31,268
#1	ultrasound OR sonography OR echography OR ultrasonography	898,262

Cochrane CENTRAL (Updated on January 19, 2022)

1. ultrasound OR sonography OR echography OR ultrasonography: 48,106
2. graft size OR graft assessment: 5,931
3. anterior cruciate ligament surgery OR anterior cruciate ligament reconstruction: 2,563
4. #1 AND #2 AND #3: 11

(2 review, 7 trials, 1 editorial, 1 clinical answers)

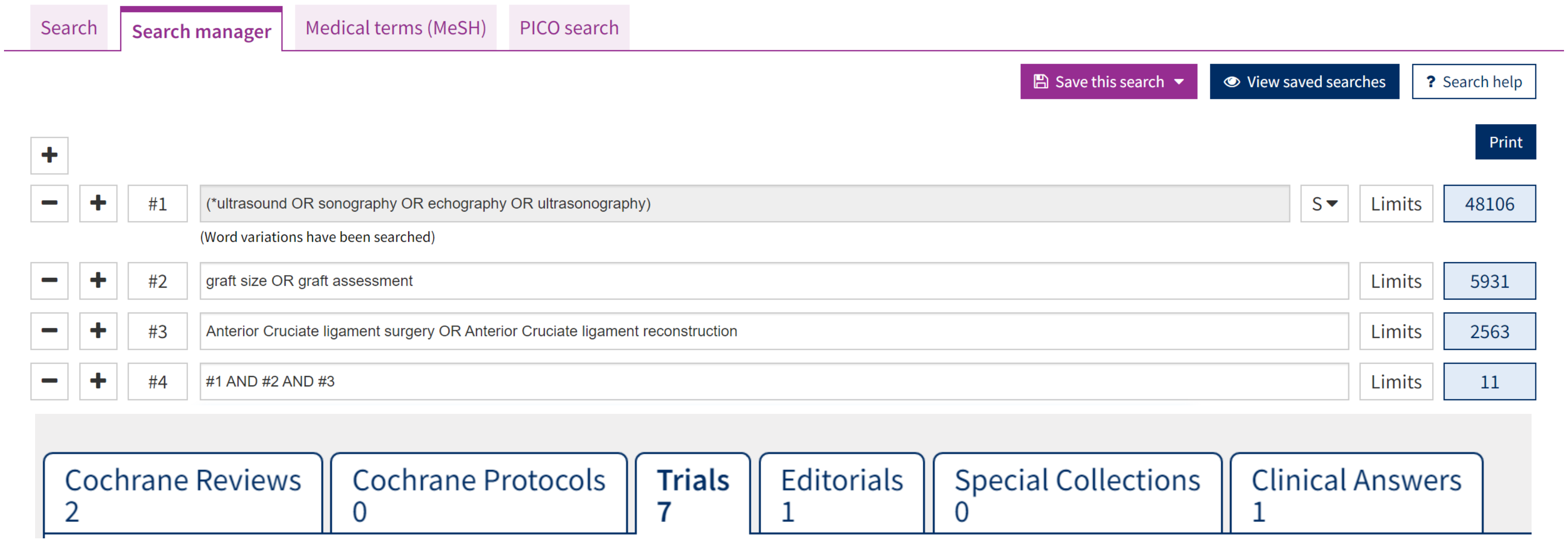

Clinical trial.gov (Updated on January 19, 2022)

Keyword:

Condition or disease: (′anterior cruciate ligament surgery′ OR ′anterior cruciate ligament reconstruction′)

Other terms: (′ultrasound′ OR ′sonography′ OR ′echography′ OR ′ultrasonography′) AND (′graft size′ OR ′graft assessment′)

78 Studies found for: **((graft size) OR (graft assessment)) AND ((anterior cruciate ligament surgery) OR (anterior cruciate ligament reconstruction)) | (Anterior Cruciate ligament surgery) OR (Anterior Cruciate ligament reconstruction)**

Completed: 20 (included)

Not completed: 58 (not included)

Web of science (Updated on January 19, 2022)

Keywords:

1. ultrasound OR sonography OR echography OR ultrasonography: too many results (>10,000)

2. graft size OR graft assessment: too many results (>10,000)

3. anterior cruciate ligament surgery OR anterior cruciate ligament reconstruction: too many results (>10,000)

4. #1 AND #2 AND #3: 17

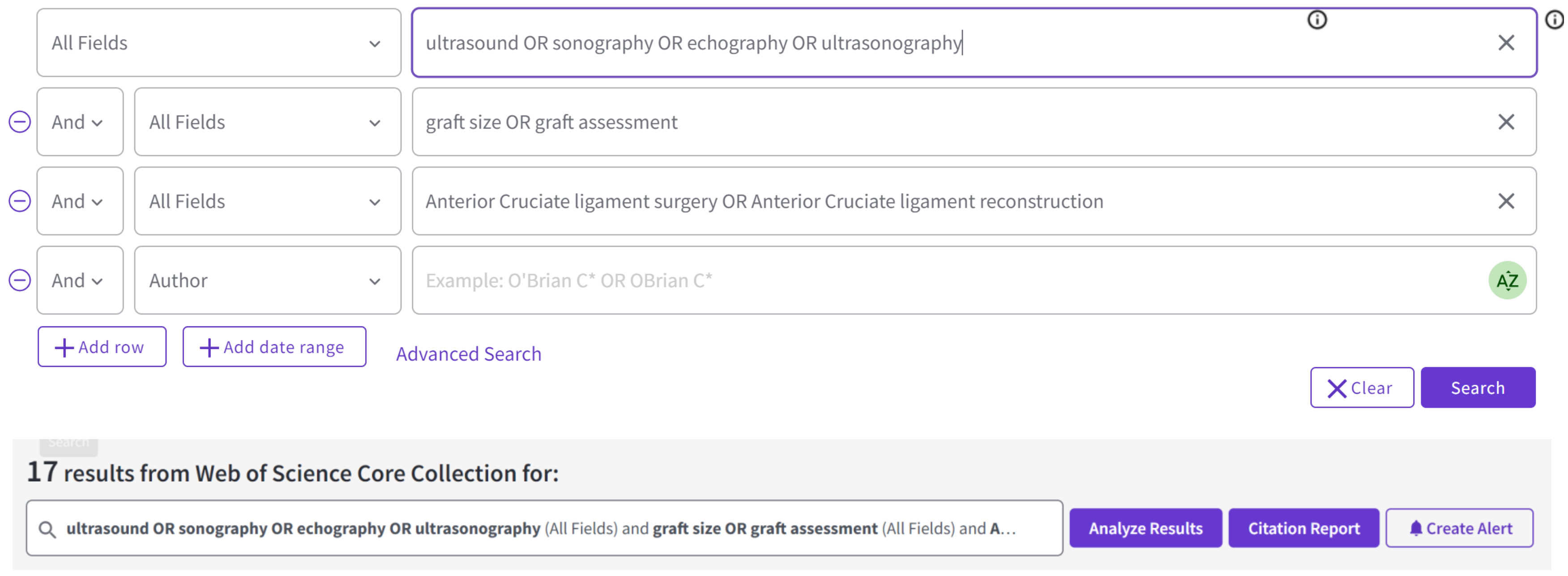

Supplement Table S1. Excluded studies and reasons

Reason	Numbers	References
Preoperational graft assessment using MRI only	25	[1-25]
Preoperational graft assessment using CT only	2	[26, 27]
Preoperational graft assessment using US but without correlation analysis with the intra-operative autograft diameter	1	[28]

Abbreviation: CT, computed tomography; MRI, magnetic resonance imaging; US, ultrasound.

References:

1. Hamada, M., et al., *Cross-sectional area measurement of the semitendinosus tendon for anterior cruciate ligament reconstruction.* Arthroscopy, 1998. **14**(7): p. 696-701.
2. Bickel, B.A., et al., *Preoperative magnetic resonance imaging cross-sectional area for the measurement of hamstring autograft diameter for reconstruction of the adolescent anterior cruciate ligament.* Arthroscopy, 2008. **24**(12): p. 1336-41.
3. Wernecke, G., et al., *Using magnetic resonance imaging to predict adequate graft diameters for autologous hamstring double-bundle anterior cruciate ligament reconstruction.* Arthroscopy, 2011. **27**(8): p. 1055-9.
4. Beyzadeoglu, T., et al., *Prediction of semitendinosus and gracilis autograft sizes for ACL reconstruction.* Knee Surg Sports Traumatol

Arthrosc, 2012. **20**(7): p. 1293-7.

5. Cobanoglu, M., et al., *Preoperative magnetic resonance imaging evaluation of semitendinosus tendon in anterior cruciate ligament reconstruction: Does this have an effect on graft choice?* Indian J Orthop, 2016. **50**(5): p. 499-504.
6. Grawe, B.M., et al., *Anterior Cruciate Ligament Reconstruction With Autologous Hamstring: Can Preoperative Magnetic Resonance Imaging Accurately Predict Graft Diameter?* Orthop J Sports Med, 2016. **4**(5): p. 2325967116646360.
7. Leiter, J., et al., *Using pre-operative MRI to predict intraoperative hamstring graft size for anterior cruciate ligament reconstruction.* Knee Surg Sports Traumatol Arthrosc, 2017. **25**(1): p. 229-235.
8. Serino, J., R. Murray, and E.H. Argintar, *Use of Magnetic Resonance Imaging to Predict Quadrupled Semitendinosus Graft Diameter in All-Inside Anterior Cruciate Ligament Reconstruction.* Orthopedics, 2017. **40**(4): p. e617-e622.
9. Zakko, P., et al., *Can we predict the size of frequently used autografts in ACL reconstruction?* Knee Surg Sports Traumatol Arthrosc, 2017. **25**(12): p. 3704-3710.
10. Ashford, W.B., et al., *Predicted quadriceps vs. quadrupled hamstring tendon graft size using 3-dimensional MRI.* Knee, 2018. **25**(6): p. 1100-1106.
11. Corey, S., et al., *Correlation of intra-operative hamstring autograft size with pre-operative anthropometric and MRI measurements.* J Orthop, 2018. **15**(4): p. 988-991.
12. Ilahi, O.A., et al., *Estimating Lengths of Semitendinosus and Gracilis Tendons by Magnetic Resonance Imaging.* Arthroscopy, 2018. **34**(8): p. 2457-2462.
13. Hodges, C.T., et al., *The medial epicondyle of the distal femur is the optimal location for MRI measurement of semitendinosus and gracilis tendon cross-sectional area.* Knee Surg Sports Traumatol Arthrosc, 2019. **27**(11): p. 3498-3504.
14. Hollnagel, K., et al., *Prediction of Autograft Hamstring Size for Anterior Cruciate Ligament Reconstruction Using MRI.* Clin Orthop Relat Res, 2019. **477**(12): p. 2677-2684.
15. Vardiabasis, N., et al., *Can We Accurately Predict the Quadruple Hamstring Graft Diameter From Preoperative Magnetic Resonance Imaging?* Orthop J Sports Med, 2019. **7**(3): p. 2325967119834504.

16. Oliva Moya, F., et al., *Can we predict the graft diameter for autologous hamstring in anterior cruciate ligament reconstruction?* Rev Esp Cir Ortop Traumatol (Engl Ed), 2020. **64**(3): p. 145-150.
17. Pérez-Mozas, M., et al., *Preoperative prediction of autologous hamstring graft diameter in anterior cruciate ligament reconstruction.* Rev Esp Cir Ortop Traumatol (Engl Ed), 2020. **64**(5): p. 310-317.
18. Thwin, L., et al., *Pre-operative MRI measurements versus anthropometric data: Which is more accurate in predicting 4-stranded hamstring graft size in anterior cruciate ligament reconstruction?* Asia Pac J Sports Med Arthrosc Rehabil Technol, 2020. **22**: p. 5-9.
19. Heijboer, W.M.P., et al., *Predictive Factors for Hamstring Autograft Diameter in Anterior Cruciate Ligament Reconstruction.* J Knee Surg, 2021. **34**(6): p. 605-611.
20. Partan, M.J., et al., *Predicting Autologous Hamstring Graft Diameter in the Pediatric Population Using Preoperative Magnetic Resonance Imaging and Demographic Data.* Am J Sports Med, 2021. **49**(6): p. 1482-1491.
21. Sherman, B., K. Kwan, and J. Schlechter, *Magnetic Resonance Imaging Predictive Model Determines Hamstring Autograft Size for Anterior Cruciate Ligament Reconstruction in Patients Under 18 Years Old.* Arthrosc Sports Med Rehabil, 2021. **3**(3): p. e715-e720.
22. Chang, C.B., S.C. Seong, and T.K. Kim, *Preoperative magnetic resonance assessment of patellar tendon dimensions for graft selection in anterior cruciate ligament reconstruction.* Am J Sports Med, 2009. **37**(2): p. 376-82.
23. Goldstein, J.L., et al., *Avoiding mismatch in allograft anterior cruciate ligament reconstruction: correlation between patient height and patellar tendon length.* Arthroscopy, 2010. **26**(5): p. 643-50.
24. Baghdadi, S., et al., *Quadriceps Tendon Autograft in Pediatric ACL Reconstruction: Graft Dimensions and Prediction of Size on Preoperative MRI.* Orthop J Sports Med, 2021. **9**(12): p. 23259671211056678.
25. Gagliardi, A.G., et al., *Prediction of quadriceps tendon-patellar bone autograft diameter in adolescents with 2-dimensional magnetic resonance imaging and anthropometric measures.* Skeletal Radiol, 2021.
26. Yasumoto, M., et al., *Predictive value of preoperative 3-dimensional computer tomography measurement of semitendinosus tendon harvested for anterior cruciate ligament reconstruction.* Arthroscopy, 2006. **22**(3): p. 259-64.
27. Truong, P.N., et al., *Preoperative Determination of the Size of the Semitendinosus and Gracilis Tendon by Multidetector Row CT Scanner*

for Anterior Cruciate Ligament Reconstruction. J Knee Surg, 2021.

28. Seijas, R., et al., *Sonographic Measurement of the Patellar Tendon Should Predict Autograft Bone Patellar Tendon Bone (BPTB) Size: Comparison of Anatomical and Clinical Findings.* J Invest Surg, 2020. **33**(7): p. 621-626.

Supplement Figure S1. Funnel plot for ultrasound measurements of the donor tendons in prediction of the autograft size among the included studies.

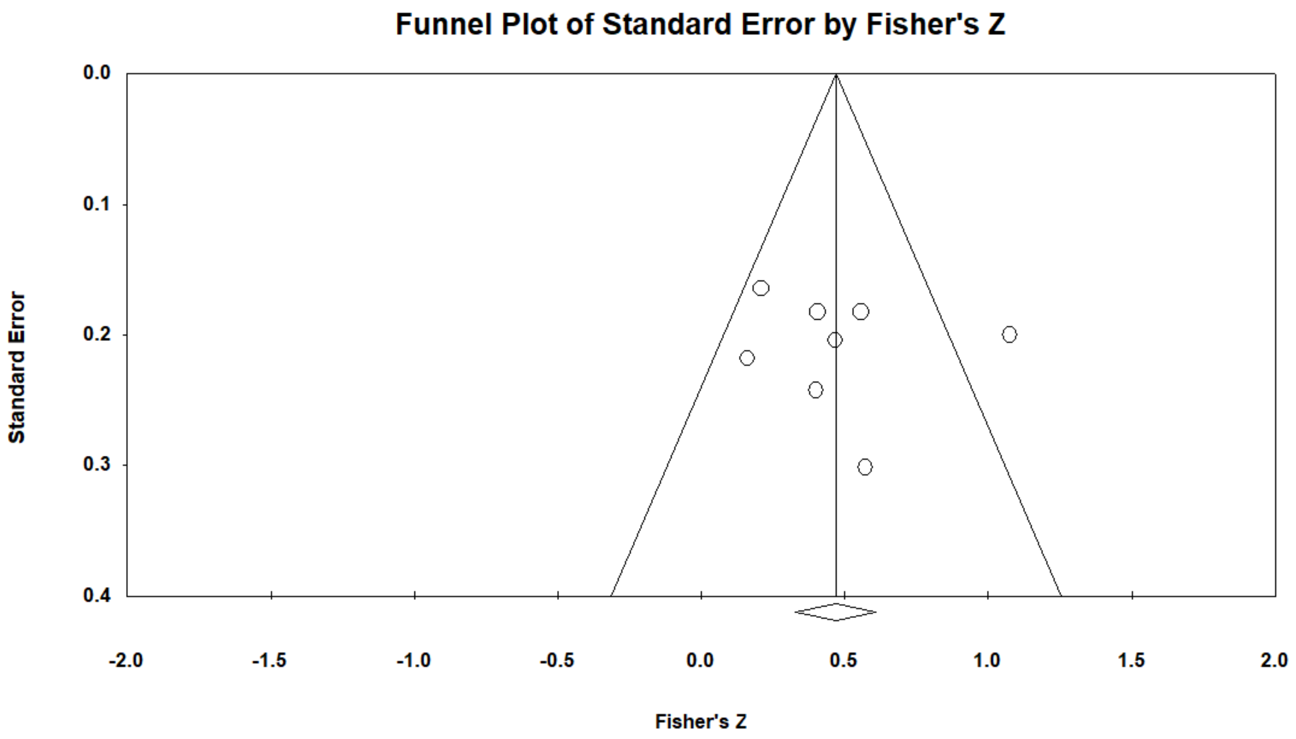

Supplement Figure S2. The summary receiver operating curve (SROC) curve of ultrasound imaging for predicting size inadequacy of the autografts. Abbreviations: AUC, area under the curve; SENS, sensitivity; SPEC, specificity; SROC, summary receiver operating curve.

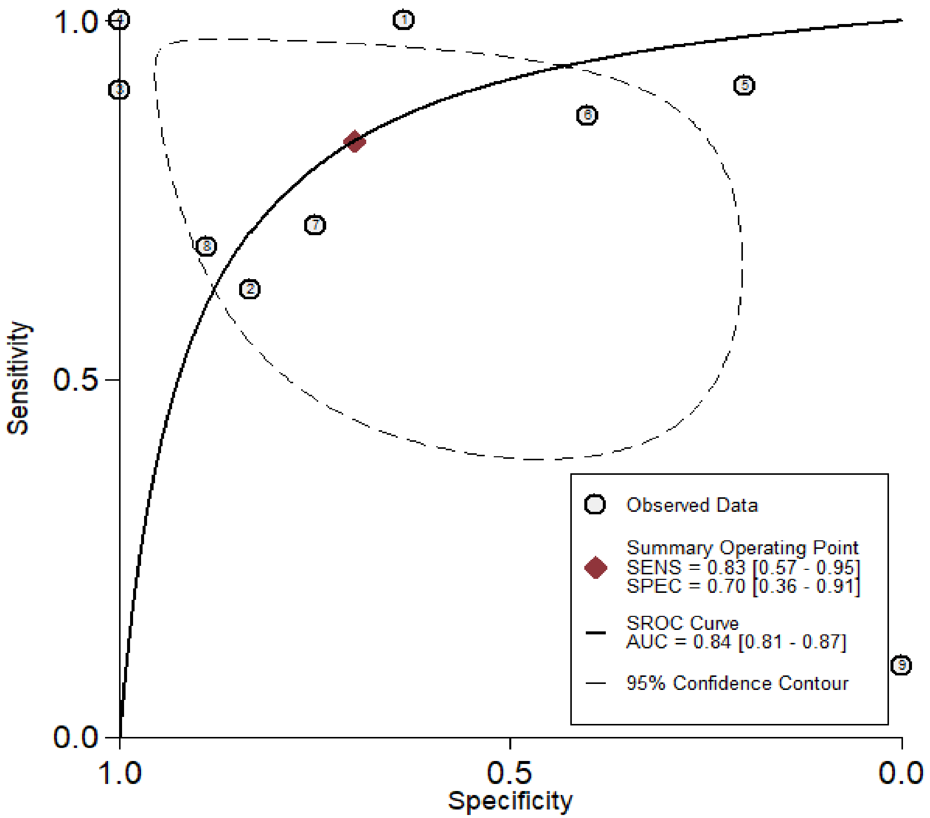

Supplement Figure S3. Deek's funnel plot for the assessment of potential publication bias of the included studies. Abbreviation: ESS, effective sample size.

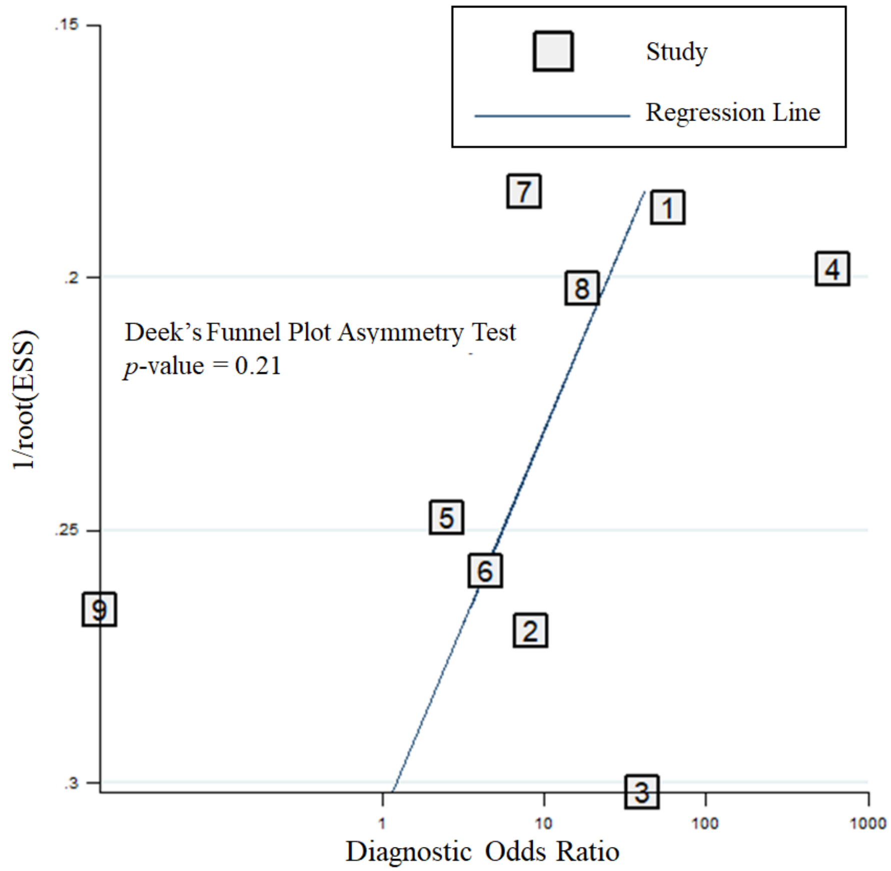

Supplement PRISMA Checklist

Section and Topic	Item #	Checklist item	Location where item is reported
**TITLE**			
Title	1	Identify the report as a systematic review.	1
**ABSTRACT**			
Abstract	2	See the PRISMA 2020 for Abstracts checklist.	1
**INTRODUCTION**			
Rationale	3	Describe the rationale for the review in the context of existing knowledge.	1-2
Objectives	4	Provide an explicit statement of the objective(s) or question(s) the review addresses.	2
**METHODS**			
Eligibility criteria	5	Specify the inclusion and exclusion criteria for the review and how studies were grouped for the syntheses.	2-3
Information sources	6	Specify all databases, registers, websites, organisations, reference lists and other sources searched or consulted to identify studies. Specify the date when each source was last searched or consulted.	2
Search strategy	7	Present the full search strategies for all databases, registers and websites, including any filters and limits used.	2 Supplementary method
Selection process	8	Specify the methods used to decide whether a study met the inclusion criteria of the review, including how many reviewers screened each record and each report retrieved, whether they worked independently, and if applicable, details of automation tools used in the process.	3
Data collection process	9	Specify the methods used to collect data from reports, including how many reviewers collected data from each report, whether they worked independently, any processes for obtaining or confirming data from study investigators, and if applicable, details of automation tools used in the process.	3
Data items	10a	List and define all outcomes for which data were sought. Specify whether all results that were compatible with each outcome domain in each study were sought (e.g. for all measures, time points, analyses), and if not, the methods used to decide which results to collect.	3
	10b	List and define all other variables for which data were sought (e.g. participant and intervention characteristics, funding sources). Describe any assumptions made about any missing or unclear information.	NA
Study risk of bias assessment	11	Specify the methods used to assess risk of bias in the included studies, including details of the tool(s) used, how many reviewers assessed each study and whether they worked independently, and if applicable, details of automation tools used in the process.	3

Effect measures	12	Specify for each outcome the effect measure(s) (e.g. risk ratio, mean difference) used in the synthesis or presentation of results.	3
Synthesis methods	13a	Describe the processes used to decide which studies were eligible for each synthesis (e.g. tabulating the study intervention characteristics and comparing against the planned groups for each synthesis (item #5)).	3
	13b	Describe any methods required to prepare the data for presentation or synthesis, such as handling of missing summary statistics, or data conversions.	3
	13c	Describe any methods used to tabulate or visually display results of individual studies and syntheses.	3
	13d	Describe any methods used to synthesize results and provide a rationale for the choice(s). If meta-analysis was performed, describe the model(s), method(s) to identify the presence and extent of statistical heterogeneity, and software package(s) used.	3-4
	13e	Describe any methods used to explore possible causes of heterogeneity among study results (e.g. subgroup analysis, meta-regression).	NA
	13f	Describe any sensitivity analyses conducted to assess robustness of the synthesized results.	4
Reporting bias assessment	14	Describe any methods used to assess risk of bias due to missing results in a synthesis (arising from reporting biases).	4
Certainty assessment	15	Describe any methods used to assess certainty (or confidence) in the body of evidence for an outcome.	4
**RESULTS**			
Study selection	16a	Describe the results of the search and selection process, from the number of records identified in the search to the number of studies included in the review, ideally using a flow diagram.	4<br>Figure 1.
	16b	Cite studies that might appear to meet the inclusion criteria, but which were excluded, and explain why they were excluded.	4<br>supplementary Table S1.
Study characteristics	17	Cite each included study and present its characteristics.	4<br>Table 1.
Risk of bias in studies	18	Present assessments of risk of bias for each included study.	7-8<br>Table 2.
Results of individual studies	19	For all outcomes, present, for each study: (a) summary statistics for each group (where appropriate) and (b) an effect estimate and its precision (e.g. confidence/credible interval), ideally using structured tables or plots.	4<br>Figure 2.<br>Figure 3.
Results of syntheses	20a	For each synthesis, briefly summarise the characteristics and risk of bias among contributing studies.	7-12<br>Table 2
	20b	Present results of all statistical syntheses conducted. If meta-analysis was done, present for each the summary estimate and its precision (e.g.	9-12

		confidence/credible interval) and measures of statistical heterogeneity. If comparing groups, describe the direction of the effect.	
	20c	Present results of all investigations of possible causes of heterogeneity among study results.	NA
	20d	Present results of all sensitivity analyses conducted to assess the robustness of the synthesized results.	9-12
Reporting biases	21	Present assessments of risk of bias due to missing results (arising from reporting biases) for each synthesis assessed.	Supplementary figure S1. figure S3.
Certainty of evidence	22	Present assessments of certainty (or confidence) in the body of evidence for each outcome assessed.	9-12
**DISCUSSION**			
Discussion	23a	Provide a general interpretation of the results in the context of other evidence.	12
	23b	Discuss any limitations of the evidence included in the review.	13
	23c	Discuss any limitations of the review processes used.	13
	23d	Discuss implications of the results for practice, policy, and future research.	13
**OTHER INFORMATION**			
Registration and protocol	24a	Provide registration information for the review, including register name and registration number, or state that the review was not registered.	14
	24b	Indicate where the review protocol can be accessed, or state that a protocol was not prepared.	14
	24c	Describe and explain any amendments to information provided at registration or in the protocol.	NA
Support	25	Describe sources of financial or non-financial support for the review, and the role of the funders or sponsors in the review.	14
Competing interests	26	Declare any competing interests of review authors.	14
Availability of data, code and other materials	27	Report which of the following are publicly available and where they can be found: template data collection forms; data extracted from included studies; data used for all analyses; analytic code; any other materials used in the review.	14

From: Page MJ, McKenzie JE, Bossuyt PM, Boutron I, Hoffmann TC, Mulrow CD, et al. The PRISMA 2020 statement: an updated guideline for reporting systematic reviews. BMJ 2021;372:n71. doi: 10.1136/bmj.n71
